# Examining the psychometric properties of the Chinese Behavioral Regulation in Exercise Questionnaire-3: A bi-factor approach

**DOI:** 10.1371/journal.pone.0265004

**Published:** 2022-03-07

**Authors:** Yan Luo, Elizabeth M. Mullin, Kathleen T. Mellano, Yanru Sha, Cheng Wang

**Affiliations:** 1 Department of Physical Education and Health Education, Springfield College, Springfield, MA, United States of America; 2 Department of Exercise Science and Athletic Training, Springfield College, Springfield, MA, United States of America; 3 Institute of Physical Education, Ningxia University, Yinchuan, Ningxia, China; 4 Department of Physical Education, Northwestern Polytechnical University, Xi’an, Shaanxi, China; Chinese Academy of Medical Sciences and Peking Union Medical College, CHINA

## Abstract

The Behavioral Regulation in Exercise Questionnaire (BREQ) was revised to its third iteration (BREQ-3) and has been widely used to measure different types of exercise motivation, including amotivation, external regulation, introjected regulation, identified regulation, integrated regulation, and intrinsic motivation. However, the Chinese version has not been similarly revised. The aim of this study was to develop and examine the psychometric properties of the Chinese BREQ-3 using alternative structural equation models. Specifically, this study aimed to translate the English BREQ-3 into Chinese to examine the best representation of the factor configuration of Chinese BREQ-3, measurement invariance for the best-fitted model, and the concurrent validity evidence and reliability for the Chinese BREQ-3. Undergraduate students (*N* = 825) from mainland China completed a battery of online questionnaires. After including two general motivation factors (controlled motivation and autonomous motivation), we discovered that the majority of items on the identified regulation, integrated regulation, and intrinsic motivation subscales no longer loaded on or had very low loadings on their specific factors, implying that these items essentially represent a unidimensional construct. Invariance testing supported the comparison between latent factor means across gender based on the bi-factor exploratory structural equation model (BESEM). Concurrent validity evidence was found for amotivation, controlled motivation, and autonomous motivation. The hierarchical omega, explained common variance (ECV), item explained common variance (I_ECV), and percentage of uncontaminated correlations (PUC) indicated that the external regulation and introjected regulation subscales had a multidimensional structure, while the identified regulation, integrated regulation, and intrinsic motivation subscales had a unidimensional structure (autonomous motivation). We advocate calculating amotivation, external regulation, introjected regulation, and a single autonomous motivation (excluding item 19) score when utilizing the Chinese BREQ-3.

## Introduction

The current conceptualization and assessment of motivation is informed by self-determination theory (SDT), which posits human behaviors to achieve particular goals can be explained by different types of motivation, including amotivation, external regulation, introjected regulation, identified regulation, integrated regulation, and intrinsic motivation [1]. These motivational types have been used to explain a variety of human actions, including work [2], teaching [3], learning [4], and video gaming [5]. In the area of exercise, the same collection of motivational types has been utilized to examine the impact of motivation on physical activity involvement [6–9]. Grounded in SDT, the Perceived Locus of Causality Questionnaire [10], Behavioral Regulation in Exercise Questionnaire (BREQ [11]), and Behavioral Regulation in Sport Questionnaire [12] have been developed, updated into more comprehensive versions over the years, and translated to other languages to expand use. The adoption of those instruments has led to theoretically informed evaluation of the relationship between motivation and physical activity across many countries. While the original BREQ has been revised into the third version(BREQ-3 [13, 14]), no comparable Chinese BREQ-3 has been developed. An up-to-date international adoption of an instrument can assist researchers in collecting data in different cultural settings as well as compare findings across populations. Hence, it is imperative to develop a corresponding Chinese BREQ-3 with promising psychometric attributes.

### Self-determination theory

SDT postulates that human motivation can be categorized into three broad taxonomies: amotivation, extrinsic motivation, and intrinsic motivation [1, 15, 16]. Amotivation pertains to individuals with the absence of intention to behave and represents unmotivated self-regulation or non-regulation [1]. Extrinsic motivation refers to an instrumentally manipulated and goal-oriented incentive to behave and depends on outcomes that are separatable from the action [1]. There are four forms of extrinsic motivation: external regulation, introjected regulation, identified regulation, and integrated regulation, representing different degrees of self-determination or autonomy [16]. External regulation is the least autonomous and most controlling form of extrinsic motivation [16]. Introjected regulation is a partially internalized form of self-regulation. Individuals with this form of regulation tend to participate in ego-oriented activities, indicating this type is more controlling rather than autonomous. Identified regulation refers to a cognitive acceptance of the values of action to achieve a preferable outcome [16]. Integrated regulation refers to the process of assimilation of identified regulation such that engaging in the behavior is completely consistent with one’s sense of self [16]. It is the most autonomous form of regulation that is extrinsically motivated. The only innately motivated type of motivation is intrinsic motivation, which refers to a natural incentive to act because of the inner satisfaction derived from that behavior and is purely self-determined [16]. In other words, when individuals are intrinsically motivated, they autonomously and freely involve themselves in an activity because of their own interest and ongoing enjoyment of doing it. Although types of motivation are conceptually distinct, they are hypothesized to be correlated and fall on the same continuum with various degrees of self-determination ordered as: amotivation (least autonomous), external regulation, introjected regulation, identified regulation, integrated regulation, and intrinsic motivation (most autonomous [16]). While the classical taxonomy of motivational types is valid and broadly used, external regulation and introjected regulation are considered as controlled motivation, whereas identified regulation and integrated regulation, in conjunction with intrinsic motivation, are classified as autonomous motivation. As a result, a revised taxonomy of motivation has been suggested, emphasizing the distinction between amotivation, controlled motivation, and autonomous motivation [17].

### The development of BREQ

BREQ was initially developed by Mullan and colleagues [11] to assess individuals’ levels of exercise motivation grounded in SDT. After removing the statistically problematic amotivation subscale, a four-factor model, consisting of external regulation, introjected regulation, identified regulation, and intrinsic motivation factors demonstrated an acceptable degree of model fit [11]. Although some validity evidence was observed for BREQ, the exclusion of the amotivation dimension remained incongruent with the taxonomy of motivation hypothesized in SDT.

To make up the dimensional gap between BREQ and SDT, BREQ-2 was developed [18], featuring the inclusion of the dimension of amotivation. An excellent model fit to the data was observed (*χ*^2^(125) = 136.49, p =.23, RMSEA = 0.02, CFI = 0.95, NNFI = 0.94, SRMR = 0.05) for a group of elderly adults. Internal consistency was also reported at acceptable levels. Several researchers have since translated the BREQ-2 into different languages and have focused on the examination of various validity evidence on this scale. The original BREQ-2 has demonstrated satisfactory construct validity evidence in English [19], Greek [20], Portuguese [21], and Chinese [22, 23]. However, other studies revealed the instability of BREQ-2 when applying it to distinct cultures [24–26]. After removing item 17, recategorizing item 8 and item 18 into identified regulation and intrinsic regulation respectively, the Spanish BREQ-2 exhibited an adequate fit to the data (*χ*^2^(125) = 2.15, CFI = 0.92, TLI = 0.90, RMSEA = 0.06, SRMR = 0.05) for a group of active exercisers [26]. After removing item 17, the hypothesized hierarchical model of the Portuguese BREQ-2 showed an acceptable level of model fit (*χ*^2^(125) = 221.70, p <.001, SRMR = 0.06, NNFI = 0.90, CFI = 0.92, RMSEA = 0.04) in which external regulation and introjected regulation were regressed on controlled motivation, whereas identified and intrinsic motivation were regressed on autonomous motivation [25]. After dropping item 17 and combining identified and intrinsic motivation into autonomous motivation, the modified 3-factor Chinese BREQ-2 (external, introjected, and autonomous motivation) demonstrated an excellent model fit to the data (*χ*^2^(74) = 117.57, CFI, GFI, NFI, and TLI >0.95, RMSEA = 0.05) among a group of middle and high school students [24].

Although BREQ-2 addressed continuum of behavior regulation from amotivation to intrinsic motivation and demonstrated evidence of validity in various settings, there remained a conceptual discrepancy between SDT and BREQ-2 in terms of the dimensions of self-regulation. SDT divides extrinsic motivation into four types: external regulation, introjected regulation, identified regulation, and integrated regulation [16]. In BREQ-2, the integrated dimension of behavior regulation was excluded due to its definitional similarity to intrinsic motivation [18]. While both represent a sense of volition, the integrated and intrinsic motivations differ in that the former is still influenced by external forces, whereas the latter is influenced solely by internal pleasure [16].

These theoretical discrepancies considered, a new BREQ instrument in English was developed which included the integrated regulation subscale but omitted the amotivation subscale [27]. The construct validity evidence of the five-factor model was confirmed by model fit indices indicating a good model fit (*χ*^2^(142) = 253.82, p <.01, CFI = 0.93; IFI = 0.93, RMSEA = 0.09). The new integrated regulation subscale demonstrated satisfactory concurrent and predictive validity evidence. González et al. [14] expanded on Wilson’s [27] and Markland and Tobin’s work [18] by developing Spanish BREQ-3, featuring the inclusion of both amotivation and integrated regulation subscales. The new Spanish BREQ-3 showed satisfactory validity and reliability evidence among adult exercisers, and measurement invariance was stablished across gender and age [14]. By using a similar manner, Cid et al. [13] developed a Portuguese version of BREQ-3, following the removal of one item from each subscale, the Portuguese BREQ-3 demonstrated satisfactory construct validity evidence (*χ*^2^(120) = 331.86, p <.001, SRMR = 0.06, NNFI = 0.91, CFI = 0.93, RMSEA = 0.06) with evidence of factorial invariance across gender. The English BREQ-3 is also available online for download [28]. To our best knowledge, no previous study has examined the validity evidence for English BREQ-3.

Zhong and Wang [9] developed a Chinese version of BREQ-3 by merging the integrated regulation scale [29] into BREQ-2, which demonstrated an acceptable model fit (*χ*^2^(194)= 565.46, p <.001, CFI = 0.90; TLI = 0.88; RMSEA = 0.06). However, the development of integrated regulation Subscale of the English version of BREQ-3 was adapted from the work of Pelletier [30] rather than Mclachlan and colleagues [29], resulting in an integrated regulation subscale in the Chinese version of the BREQ-3 that is not aligned with the English version. Specifically, the English version contains items: “I exercise because it is consistent with my life goals; I consider exercise part of my identity; I consider exercise a fundamental part of who I am; and I consider exercise consistent with my values”. The Chinese version includes items: “it is consistent with my values, goals and aims in life; it is essential to my identity and sense of self; it is genuinely part of me; and doing exercise and being myself are inseparable”. While the first three questions are fairly similar in meaning, the last question is phrased noticeably different. This discrepancy may lead to misunderstanding and reduced ability to make comparisons. Sperber et al. [31] argued that failing to maintain the meaning of the original items might result in conclusions that appear culturally distinct but are actually the result of instrument inequivalence. Hence, using congruent integrated regulation subscales for both English and Chinese BREQ-3 is necessary to ensure cross-cultural equivalence of BREQ-3.

### Limitations of confirmatory factor analysis

**Fig 1:**
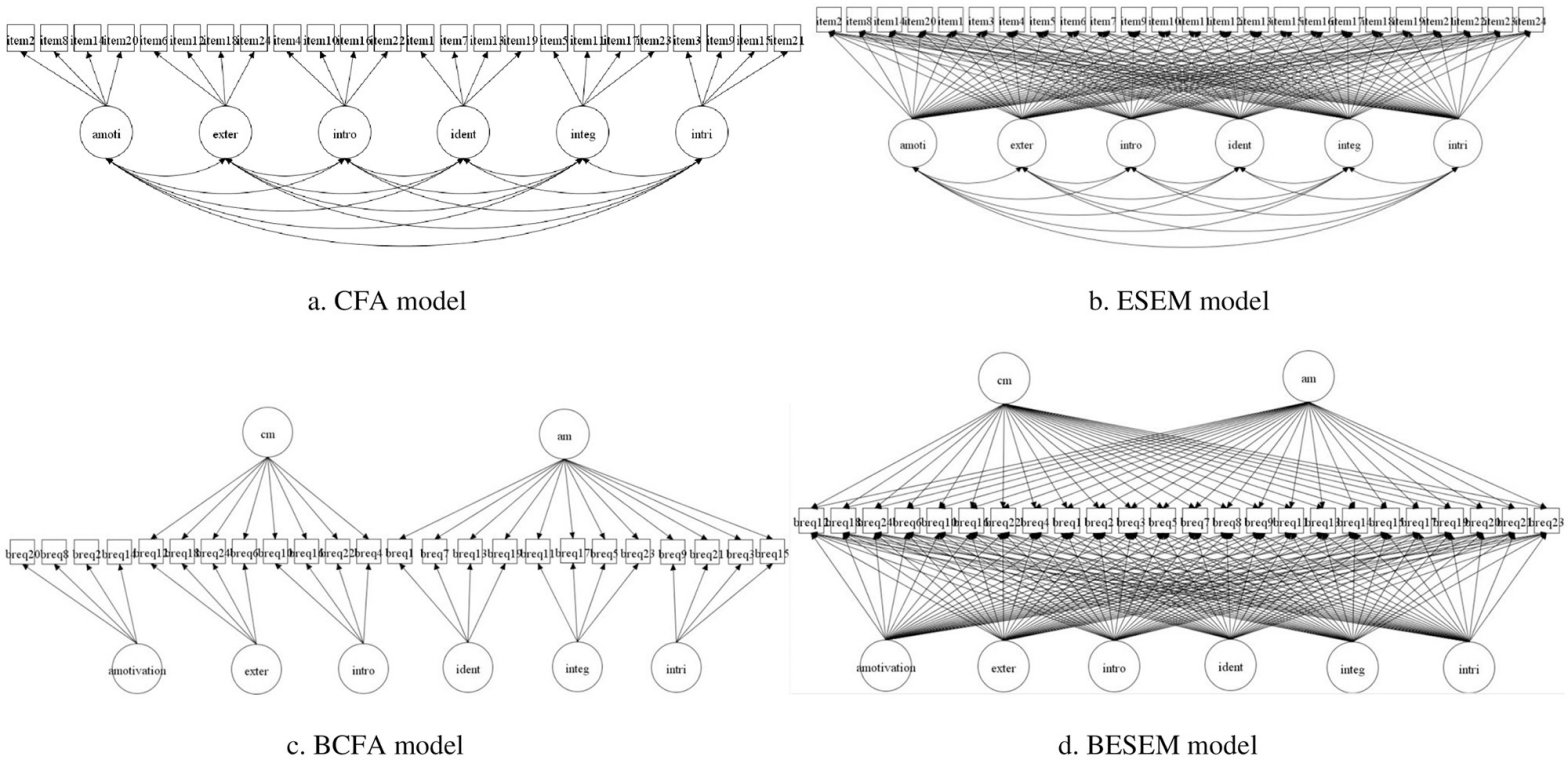
Graphical illustration of four alternative structural equation models. cm = controlled motivation; am = autonomous motivation; amoti = amotivation; exter = external regulation; intro = introjected regulation; ident = identified regulation; integ = integrated regulation; intri = intrinsic motivation; CFA = confirmatory factor analysis; ESEM = exploratory structural equation modeling; BCFA = bi-factor confirmatory factor analysis; BESEM = bi-factor exploratory structural equation modeling.

Previous researchers have predominantly relied on confirmatory factor analysis (CFA) to examine the theoretically assumed dimensionality of BREQ-3 [13, 27]. Although this statistical approach has been widely used in the examination of the construct validity evidence for psychological instrument in various contexts, it has several limitations. CFA implicitly assumes that items loading on one latent factor have no effect on other factors (cross-loadings) and thus coerce them to 0. This assumption may lead to a biased parameter estimation [32]. In addition, the exclusion of cross-loading estimation in a CFA model may result in an overestimation of the correlation between latent factors as the only way to represent the association between indicators and other factors is to inflate the factor correlations [33]. To overcome these limitations, exploratory structural equation modeling (ESEM) was developed to model data based on CFA with advantages to estimate cross-loadings between indicators and latent factors, thus providing a more realistic parameter estimation [34]. Another improvement on the traditional CFA model is the use of bi-factor modeling approach. Bi-factor CFA (BCFA) assumes that the covariances among all indicators can be explained by a general latent factor and the remaining covariances can be further explained by specific latent factors [35]. A prominent advantage of BCFA is the independence of latent factors. In other words, all of the latent factors estimated from a BCFA model are assumed to be uncorrelated, thus making results more interpretable [36]. ESEM has also been integrated with bi-factor modeling into a broader statistical framework called bi-factor exploratory structural equation modeling (BESEM [37]), which comprises the advantages of both BCFA and ESEM. To align with SDT, the present study used two global factors to model controlled (external regulation and introjected regulation) and autonomous motivation (identified regulation, integrated regulation, and intrinsic motivation) and used six specific factors to model amotivation, external regulation, introjected regulation, identified regulation, integrated regulation, and intrinsic motivation. A previous study found redundant information shared by specific motivation factors in the Chinese BREQ-2 [24], therefore a bi-factor approach will provide evidence to determine if specific motivational types provide additional information in explaining the common variance shared among items beyond the common variance explained by the two global factors. It is recommended to compare different structural equation models as it is preferred to select a more parsimonious factor architecture rather than a more complex one when assessed models exhibited parallel model fit [33]. All four models are presented in Fig 1.

In light of the aforementioned limitations, this study aimed to investigate the psychometric properties of the Chinese BREQ-3. Specifically, this study aimed to: (a) translate the English BREQ-3 into Chinese; (b) examine the best representation of the factor configuration of Chinese BREQ-3 using CFA, BCFA, ESEM, and BESEM models; (c) examine measurement invariance for the best-fitted model across gender, and (d) test the concurrent validity evidence and reliability for the best-fitted model by correlating types of motivation with theoretically related psychological constructs, such as positive and negative affect [38] and basic psychological needs [20].

## Materials and methods

### Participants

Undergraduate college students (*N* = 825) from several public universities in the mainland of China participated in this study. Student athletes were excluded to ensure the external validity of this study. The participants’ average age was 19.72 (±1.25). Respondents (*n* = 3, 0.36%) who did not report gender information were excluded from this study. Table 1 shows the background information for the participants.

**Table 1:** Background information for the participants (*N* = 822).

Variable		Frequency	Percentage
*Sex*			
	Men	287	34.9%
	Women	535	65.1%
*College* *year*			
	Freshman	399	48.5%
	Sophomore	394	47.9%
	Junior	19	2.3%
	Senior	10	1.2%
*Types* *of* *exercise* (multiple response)			
	Aerobic	671	81.6%
	Non-aerobic	125	15.2%
	Sport	366	44.5%
	Gym	83	10.1%
*Exercise* *frequency* *per* *month*			
	0 time	10	1.2%
	1-5 time(s)	301	36.6%
	6-10 times	209	25.4%
	11-15 times	100	12.2%
	>16 times	185	22.5%
	Did not respond	17	2.1%
*Exercise* *time* *per* *session*			
	<1 hour	510	62.0%
	1-2 hour(s)	272	33.1%
	2-3 hours	22	2.7%
	>3 hours	4	0.5%
	Did not respond	14	1.7%
*Subjective* *report* *of* *intensity* *per* *session*			
	Low	370	45.0%
	Moderate	418	50.9%
	High	21	2.6%
	Did not respond	13	1.6%

#### Behavioral Regulation in Exercise Qustionnaire-3

The 24-item English version of BREQ-3 [18, 27] was translated into Chinese for this study. The instrument measures six different types of behavioral regulation with four indicators under each dimension: amotivation (e.g., “I don’t see why I should have to exercise”), external regulation (e.g., “I exercise because other people say I should”), introjected regulation (e.g., “I feel guilty when I don’t exercise”), identified regulation (e.g., “It’s important to me to exercise regularly”), integrated regulation (e.g., “I exercise because it is consistent with my life goals”) and intrinsic motivation (e.g., “I exercise because it’s fun”). The score of each item was obtained from the individual’s subjective evaluation on a Likert scale ranging from 0 (not true for me) to 4 (very true for me). The score for each type of behavioral regulation was calculated by averaging the scores of the items that are under the same category. To our knowledge, no prior study has examined the validity and reliability for the English BREQ-3.

#### Chinese Positive and Negative Affect Schedule Short Form

The Chinese Positive and Negative Affect Schedule Short Form (I-PANAS-SF [38]) was used to measure participants’ positive and negative affect. The stem begins: “during exercise”. The I-PANAS-SF includes 10 items with 5 items measuring positive (e.g., “I am active”) and negative (e.g., “I am nervous”) affect (PA, NA) respectively. Each item in I-PANAS-SF was subjectively evaluated by individuals on a Likert scale ranging from 1 (never) to 5 (always). Participants indicated to what degree they agree with each item. The score for each subscale was calculated by averaging the scores of the items from the same affect subscale. The subscales showed good construct validity evidence (*χ*^2^(19) = 406.97, p <.01, CFI = 0.96; TLI = 0.92, RMSEA = 0.07, SRMR = 0.03) and internal consistency among a Chinese population (*α* = 0.81 and 0.83 for PA and NA respectively [38]).

#### Chinese Basic Psychological Needs in Exercise Scale

The Chinese Basic Psychological Needs in Exercise Scale (BPNES [39]) assessed individual basic psychological needs in exercise. The BPNES consists of 11 items measuring sense of autonomy (e.g., “The way I exercise is in agreement with my choices and interests”), competence (e.g., “I feel exercise is an activity which I do very well”), and relatedness (e.g., “I feel I have excellent communication with the people I exercise with”). Response options range from 1 (totally disagree) to 5 (very strongly agree). Participants indicated to what degree they agree with each item. The score for each subscale was calculated by averaging the scores of the items within the same subscale. The subscales of BPNES have demonstrated good construct validity evidence (*χ*^2^(41) = 144.95, p <.001, CFI = 0.95, RMSEA = 0.07, SRMR = 0.04) and internal consistency among Chinese college students (*α* = 0.75, 0.81, and 0.86 for autonomy, competence, and relatedness respectively [39]).

### BREQ-3 translation to Chinese

A combined translation approach was used to translate the English version of BREQ-3 into simplified Chinese [40]. Three bilingual students, who have been studying in the United States for two to three years independently translated the instruments. The bilingual translators included two doctoral students majoring in Physical Education and an undergraduate student majoring in Sport Management. Each individually translated instrument was scrutinized by the other two bilingual translators. Any discrepancies between translated instruments were discussed in a meeting among the translators. This procedure continued until three translators agreed with each other and constructed a final version of translated instrument. The translated instrument was then back-translated by a bilingual Chinese professor who has been living in U.S. for over 15 years. The original and back-translated versions of the instrument were assessed by two monolingual English-speaking professors in sport and exercise psychology. If any differences were identified, the monolingual professors provided three forward-translators detailed explanations of the difference between two instruments. Following discussion, the three translators modified the wording of the problematic items and the back-translator translated the adapted items into English again. The process continued until the monolingual reviewers considered the two English versions were identical. The final product of this translation process, the Chinese BREQ-3, was used in this study.

### Procedures

The study was approved by the Institutional Review Board (IRB) of the first author’s institution. Convenience sampling approach was used to recruit participants in this study. After obtaining permission to conduct this study, the researchers contacted professors in China willing to distribute the surveys through personal networks. After obtaining permission, the students taught by those professors were invited to participate in this study.

An implied consent form, Chinese translated version of BREQ-3, I-PANAS-SF, and BPNES were presented to the potential participants using Wen Juan Xing (TongdaoLiepin, Beijing), an online questionnaire management software, and the questionnaires were virtually sent to students via Wechat (Tencent, Shenzhen), an instant mobile messaging app. Students were asked to voluntarily fill out the questionnaires either at the end of a class or at their preferred time and location, depending on the professor’s preference. Completing the questionnaires took approximately 15 minutes. The finished questionnaires were automatically stored online in a password protected account and were only accessible to the primary researcher of this study. Data were downloaded to the primary researcher’s laptop encrypted with passwords for analysis purpose.

### Data analysis

R (Version 4.0.2, R Core Team, www.r-project.org) was used for data preparation and Mplus [41] was used to analyze data. Descriptive statistics for the items of BREQ-3 was computed. The Shapiro-Wilk test and the Mardia estimate of multivariate Kurtosis were used to assess univariate and multivariate normality of the item scores respectively. Items with z scores higher than 3.29 or less than -3.29 were considered outliers [42]. Robust maximum likelihood estimator (MLR) was utilized to examine the fitting of the factor structure of Chinese BREQ-3 as it provided robust fit indices with the occurrence of non-normality and outliers and was suitable for Likert scale with five or more response categories [43].

In the CFA model, items were specified to regress on a priori factor with no cross loading permitted. All factors were allowed to correlate with each other. In the BCFA model, items were specified to regress on two general factors (except for amotivation) as well as on their specific factors with no cross loading permitted. All factors were not allowed to correlate with each other. In the ESEM model, each item was allowed to regress on every factor with oblique target rotation used to make cross-loadings as close to zero as possible. All factors were allowed to correlate with each other. In the BESEM model, each item was allowed to regress on every factor with oblique target rotation used to make cross-loadings as close to zero as possible. All factors were not allowed to correlate with each other.

The following goodness of fit indices were analyzed for each of the four models: chi-square (*χ*^2^), comparative fit index (CFI [44]), Tucker-Lewis index (TLI [45]), and root mean square error of approximation (RMSEA [46]). Akaike information criterion (AIC [47]), Bayesian information criterion (BIC [48]), and Akaike’s Bayesian information criterion (ABIC [49]) were used to assess whether model fit outperformed model complexity. The cut-off values for the indication of a good model fit for those indexes are: p >.05 for *χ*^2^, CFI >0.95, TLI >0.95, RMSEA <0.06 [50], and p-close for RMSEA >.05 [51]. No rule of thumb was used to determine the adequacy of AIC, BIC, and ABIC values, but lower values generally suggest a better trade-off between model fit and model complexity [52]. A decrease in CFI less than 0.01 or an increase in RMSEA less than 0.015 between assessed models was used to test model difference [53, 54]. A multi-group model, depending which model has the best fit, was conducted to test the measurement invariance between men and women. The steps of invariance testing followed the suggestions from Putnick and Bornstein [52]. First, we fitted the best model separately to the men and women groups. We then proceeded to test for configural (no equality constraints), metric (constrain factor loadings), scalar invariance (constrain factor loadings and intercepts), and strict invariance (constrain factor loadings, intercepts, and residual variances) between groups. A decrease in CFI less than 0.01 or an increase in RMSEA less than 0.015 from less-constrained model to the more-constrained model was used as evidence of gender invariance [54]. Next, concurrent evidence of validity was assessed by regressing the scores obtained from the best model onto the scores from I-PANAS-SF and BPNES [52].

The composite reliability omega (*ω*_c_) and hierarchical omega (*ω*_h_) coefficients were calculated to assess the model-based reliability [55]. Composite omega is the ratio of the estimated true variance of the score of a measure to its total variance, thereby indicating internal consistency [56]. A composite omega value greater than 0.80 indicates that the internal consistency is satisfactory [57]. A hierarchical omega index measures the proportion of total variance that can be traced to individual differences in the general factor [56]. If the hierarchical omega is being applied to specific factors in a bi-factor model, it measures the proportion of total variance that can be traced to individual differences in the specific factors. Hierarchical omega value greater than 0.50 is indictive of satisfactory factor reliability [58]. If the hierarchical omega value is larger than 0.80, the total score should be considered unidimensional [59]. The explained common variance (ECV) was used to determine the degree to which the common variance among a set of items is explained by a general factor [60]. ECV greater than 0.85 should be regarded as essentially unidimensional. Item explained common variance (I-ECV) was used to assess the item common variance that is attributable to a general factor [60]. I-ECV greater than 0.85 indicates an item essentially reflects the general factor [60]. To determine the extent to which item correlations inform the general factor, the percentage of uncontaminated correlations (PUC) was computed. The greater the PUC, the more saturated the correlation matrix is with information useful for estimating the parameters for the general factor, and the less probable it is that the parameter estimations in a unidimensional model would be biased. When the PUC is less than 0.80, ECV is greater than 0.60, and the hierarchical omega is greater than 0.70, the existence of some multidimensionality is not severe enough to reject the instrument’s interpretation as largely unidimensional [58].

## Results

### Data screening

Prior to analysis, data screening was conducted. No missing values were found. Outliers were identified within item 8 (19), item 14 (20), item 20 (20), and the amotivation subscale (12). We decided not to delete the outliers because they resulted from the ceiling and flooring effect of the responses rather than unexplainable errors [61]. The Shapiro-Wilk test indicated that none of the item scores met univariate normality (all ps <.05). The Mardia estimate of multivariate Kurtosis indicated deviation of the item scores from multivariate normality (p <.05). Potential biases of the non-normality and outliers were compensated by using robust estimation procedure [61].

### Goodness of fit

Next, we evaluated the goodness of fit among the four competing models (Table 2). Given the large sample size, none of the models fit well based on the *χ*^2^ statistics (p <.001). According to other goodness of fit indices, the CFA model fit the data poorly. The ESEM model showed almost excellent fit. When examining the bi-factor models, the BCFA model showed poor fit and the BESEM model demonstrated excellent fit. The decrease in AIC, BIC, and ABIC values indicate that the increase in model fit outperformed the increase in complexity from ESEM to BESEM. While initial inspection of the goodness of fit indices indicated strong evidence for selecting the BESEM model over other alternatives, further examination of the parameter estimates will assist in determining the optimal structure of the Chinese BREQ-3.

**Table 2:** Goodness of fit of alternative structural equation models.

Models	*χ*^2^(df)	p	CFI	TLI	RMSEA (90% CI)	p-close	AIC	BIC	ABIC
***CFA***	1339.829 (237)	<.001	0.88	0.86	0.075 (0.071-0.079)	<.001	48103.19	48513.11	48236.84
***ESEM***	465.122 (147)	<.001	0.97	0.94	0.051 (0.046-0.057)	.332	47109.81	47943.79	47381.70
***BCFA***	1901.580 (232)	<.001	0.82	0.79	0.094 (0.090-0.097)	<.001	48711.79	49145.27	48853.11
***BESEM***	258.894 (112)	<.001	0.98	0.96	0.040 (0.034-0.046)	1.00	46939.15	47938.04	47,264.80

Table notes CFA = confirmatory factor analysis; ESEM = exploratory structural equation modeling; BCFA = bi-factor confirmatory factor analysis; BESEM = bi-factor exploratory structural equation modeling; *χ*^2^ = chi-square; df = degrees of freedom; CFI = comparative fit index; TLI = Tucker-Lewis index; RMSEA = root mean square error of approximation; CI = confidence interval; AIC = Akaike information criterion; BIC = Bayesian information criterion; ABIC = Akaike Bayesian information criterion; p-close indicates the p value for the test of close fit.

### Parameter estimates

Table 3 shows the standardized factor loadings for the CFA and ESEM models. Each item was significantly loaded on their priori-specified factors in the CFA model. In the ESEM model, item 13, 19 under the identified regulation subscale, item 3 under the intrinsic motivation subscale, and all items under the integrated regulation subscale were not significantly loaded on their intended factors. Cross-loadings of those items suggested that they were associated with adjacent or even distant factors.

**Table 3:** Standardized factor loadings for confirmatory factor analysis and exploratory structural equation models.

	CFA						ESEM					
Items	Amoti (λ)	Exter (λ)	Intro (λ)	Ident (λ)	Integ (λ)	Intri (λ)	Amoti (λ)	Exter (λ)	Intro (λ)	Ident (λ)	Integ (λ)	Intri (λ)
*BREQ*14	0.900***						**0.824** ***	0.081*	0.005	-0.080	0.094	0.074
*BREQ*2	0.638***						**0.621** ***	0.054	0.059	-0.035	-0.255***	0.089
*BREQ*20	0.846***						**0.791** ***	0.107*	-0.063	0.035	0.281***	-0.053
*BREQ*8	0.837***						**0.854** ***	0.070	0.016	0.011	-0.211***	0.119
*BREQ*12		0.686***					-0.113**	**0.843** ***	-0.016	0.013	-0.075	0.007
*BREQ*18		0.797***					0.255***	**0.469** ***	0.162**	-0.086	0.229***	-0.083
*BREQ*24		0.799***					0.189***	**0.658** ***	-0.087*	0.040	0.198***	-0.130
BREQ6		0.748***					0.024	**0.856** ***	0.009	0.095	-0.267***	-0.022
*BREQ*10			0.777***				0.089**	0.036	**0.761** ***	-0.047	-0.035	0.024
BREQ16			0.743***				0.043	0.196***	**0.603** ***	-0.222*	0.108	0.103
*BREQ*22			0.689***				-0.006	0.040	**0.538** ***	-0.105	0.179*	0.205*
*BREQ*4			0.651***				-0.022	0.005	**0.739** ***	0.224**	-0.089	-0.165*
*BREQ*1				0.712***			-0.009	-0.026	0.099	**0.668** **	0.193	-0.035
*BREQ*13				0.771***			-0.288***	0.168***	-0.069	**0.320**	0.141	0.342*
*BREQ*19				0.305***			0.017	0.159***	0.591***	**-0.082**	0.261***	-0.018
*BREQ*7				0.791***			-0.167**	0.044	-0.030	**0.403** *	0.219	0.262**
*BREQ*11					0.805***		0.004	-0.015	0.202***	0.331***	**0.176**	0.307*
*BREQ*17					0.803***		-0.028	0.012	0.129**	0.356***	**0.190**	0.304*
*BREQ*23					0.813***		-0.153***	0.056	0.085	0.253	**0.176**	0.424***
*BREQ*5					0.736***		-0.004	0.011	0.212***	0.372***	**0.154**	0.209*
BREQ15						0.873***	-0.014	-0.024	-0.010	0.061	0.079	**0.796** ***
*BREQ*21						0.859***	-0.026	-0.029	0.050	-0.007	0.119	**0.791** ***
*BREQ*3						0.669***	0.147***	-0.149***	0.141**	0.266	0.103	**0.354**
*BREQ*9						0.835***	0.059	-0.088*	0.098*	0.158	0.113	**0.607** **

Table notes Amoti = amotivation; Exter = external regulation; Intro = introjected regulation; Ident = identified regulation; Integ = integrated regulation; Intri = intrinsic motivation; CFA = confirmatory factor analysis; ESEM = exploratory structural equation modeling;

* = p <.05;

** = p <.01;

*** = p <.001;

bolded factor-loadings indicate items under the pre-specified factor.

Table 4 shows the standardized factor loadings for the BCFA and BESEM models. The factor loadings for the amotivation items were significant and high in both BCFA and BESEM model. While all the items from the external regulation and introjected regulation subscales significantly loaded on the controlled motivation factor in both the BCFA and BESEM models, all of these items also significantly loaded on their specific factors (except for item 18 in the BCFA model). While all the items from the identified regulation, integrated regulation, and intrinsic motivation subscales significantly loaded on the autonomous motivation factor, most of them did not load on their specific factors or had significant but relatively low factor loadings. The findings from the goodness of fit statistics and the parameter estimates indicate that the BESEM model should be accepted for further analysis.

**Table 4:** Standardized factor loadings for bi-factor confirmatory factor analysis and bi-factor exploratory structural equation models.

	BCFA								BESEM								
Items	CM (λ)	AM (λ)	Amoti (λ)	Exter (λ)	Intro (λ)	Ident (λ)	Integ (λ)	Intri (λ)	CM (λ)	AM (λ)	Amoti (λ)	Exter (λ)	Intro (λ)	Ident (λ)	Integ (λ)	Intri (λ)	I_ECV
*BREQ*14			0.904***						0.419***	-0.129***	**0.751** ***	0.132**	0.018	-0.118***	0.001	-0.007	
*BREQ*2			0.636***						0.147***	-0.215***	**0.561** ***	0.220***	0.159***	-0.034	-0.033	0.049	
*BREQ*20			0.843***						0.467***	-0.046	**0.742** ***	0.076	-0.057	-0.042	-0.066	-0.118*	
*BREQ*8			0.836***						0.193***	-0.203***	**0.801** ***	0.269***	0.175***	-0.010	0.038	0.043	
*BREQ*12	0.651***			0.439***					**0.478** ***	-0.015	0.128***	**0.608** ***	-0.023	-0.002	0.043	0.020	0.382
*BREQ*18	1.014***			-0.359					**0.666** ***	0.008	0.395***	**0.241** **	0.040	-0.030	-0.107*	-0.054	0.884
*BREQ*24	0.707***			0.227**					**0.564** ***	-0.051	0.364***	**0.395** ***	-0.093**	0.020	-0.001	-0.098*	0.671
*BREQ*6	0.696***			0.458***					**0.418** ***	-0.101**	0.256***	**0.691** ***	0.087**	0.023	-0.005	0.007	0.268
*BREQ*10	0.412***				0.687***				**0.502** ***	0.276***	0.181***	0.021	**0.495** ***	-0.024	0.069	0.028	0.507
*BREQ*16	0.505***				0.548***				**0.633** ***	0.211***	0.178***	0.032	**0.314** ***	-0.048	0.056	0.095**	0.803
*BREQ*22	0.279***				0.600***				**0.480** ***	0.412***	0.079***	-0.095**	**0.279** **	-0.001	0.190**	0.110	0.747
*BREQ*4	0.289***				0.618***				**0.337** ***	0.337***	0.057*	0.044	**0.576** ***	0.016	-0.059	-0.096	0.255
*BREQ*1		0.696***				0.104			-0.010	**0.728** ***	-0.039	0.022	0.122**	**0.059**	-0.005	-0.168*	0.993
*BREQ*13		0.721***				0.310***			-0.027	**0.687** ***	-0.201***	0.033	-0.021	**0.554** *	0.039	0.056	0.606
*BREQ*19		0.383***				-0.341***			0.636***	**0.327** ***	0.152***	-0.026	0.291***	**-0.032**	0.030	-0.016	0.991
*BREQ*7		0.746***				0.337***			-0.009	**0.739** ***	-0.165***	-0.027	-0.025	**0.174**	0.007	0.011	0.947
*BREQ*11		0.776***					0.329*		0.113***	**0.765** ***	-0.005	0.001	0.108**	-0.049	**0.262** *	0.011	0.895
*BREQ*17		0.780***					0.284		0.086**	**0.757** ***	-0.028	-0.004	0.053	0.032	**0.328** **	-0.010	0.842
*BREQ*23		0.827***					-0.042		0.068*	**0.754** ***	-0.121***	-0.027	0.041	0.174	**0.197** *	0.119*	0.936
*BREQ*5		0.716***					0.114		0.126**	**0.710** ***	-0.014	0.025	0.126***	-0.076	**0.136** *	-0.013	0.965
*BREQ*15		0.809***						0.315*	-0.020	**0.813** ***	-0.052**	-0.021	-0.008	0.085*	0.018	**0.325** ***	0.862
*BREQ*21		0.817***						0.229	0.051	**0.785** ***	-0.048*	-0.064*	0.010	0.096	0.116	**0.335** ***	0.846
*BREQ*3		0.620***						0.282*	0.002	**0.695** ***	0.057*	-0.023	0.112**	-0.170**	-0.130*	**0.069**	0.990
*BREQ*9		0.769***						0.374**	0.022	**0.819** ***	-0.009	-0.022	0.048	-0.110	-0.065	**0.223**	0.931
*ω_c_*									0.88	0.95	0.87	0.86	0.80	0.82	0.88	0.90	
*ω_h_*									0.63	0.92		0.40	0.33	0.06	0.08	0.07	
*ECV*									0.54	0.89							
*PUC*									0.57	0.73							

Table notes CM = controlled motivation; AM = autonomous motivation; Amoti = amotivation; Exter = external regulation; Intro = introjected regulation; Ident = identified regulation; Integ = integrated regulation; Intri = intrinsic motivation; *ω*_c_ = composite omega; *ω*_h_ = hierarchical omega; ECV = explained common variance; I_ECV = item explained common variance; PUC = percentage of uncontaminated correlations; BCFA = bi-factor confirmatory factor analysis; BESEM = bi-factor exploratory structural equation modeling;

* = p <.05;

** = p <.01;

*** = p <.001;

bolded factor-loadings indicate items under the pre-specified factor.

### Factor correlations

Factor correlations for the CFA and ESEM models were calculated and are reported in Table 5. Overall, the factor correlations in the ESEM model are substantially lower than those in the CFA model. For both model types, factors generally correlated most closely with the most proximal factor. More distal factors demonstrated non-significant or negative relationships.

**Table 5:** Factor correlations for the confirmatory factor analysis and exploratory structural equation models.

**Factors**	**1**.	**2**.	**3**.	**4**.	**5**.	**6**.
***1.Amoti***	-	0.77***	0.42***	-0.31***	-0.15**	-0.19***
***2.Exter***	0.59***	-	0.54***	-0.09	<0.01	-0.09
***3.Intro***	0.26***	0.41***	-	0.40***	0.55***	0.43***
***4.Ident***	-0.29**	-0.07	0.33*	-	0.93***	0.90***
***5.Integ***	0.02	0.14**	0.44***	0.29***	-	0.92***
***6.Intri***	-0.27***	-0.02	0.39***	0.73***	0.44	-

Table notes Amoti = amotivation; Exter = external regulation; Intro = introjected regulation; Ident = identified regulation; Integ = integrated regulation; Intri = intrinsic motivation;

* = p <.05;

** = p <.01;

*** = p <.001.

Upper diagonal shows the factor correlations in the confirmatory factor analysis model. Lower diagonal shows the factor correlations in the exploratory structural equation model.

### Measurement invariance

Table 6 shows the measurement invariance test across men and women. CFI, TLI, and RMSEA values represented an excellent fit of the BESEM model for both men and women groups. Comparing the configural, metric, scalar, and strict invariance tests, there was no substantial decrease in the CFI values or increase in RMSEA values.

**Table 6:** Measurement invariance testing between men and women.

Models	*χ*^2^(df)	p	CFI	TLI	RMSEA (90%CI)	ΔCFI	ΔRMSEA
*Men*	176.559 (112)	<.001	0.98	0.96	0.045 (0.032-0.057)		
*Women*	266.999 (112)	<.001	0.97	0.94	0.051 (0.043-0.059)		
*Configural* *invariance*	429.410 (224)	<.001	0.98	0.95	0.047 (0.040-0.054)		
*Metric* *invariance*	682.448 (352)	<.001	0.97	0.95	0.048 (0.042-0.053)	-0.01	0.001
*Scalar* *invariance*	626.387 (376)	<.001	0.97	0.96	0.040 (0.035-0.046)	0	-0.008
*Strict* *invariance*	645.234 (400)	<.001	0.97	0.96	0.039 (0.033-0.044)	0	-0.001

Table notes *χ*^2^ = chi-square; df = degrees of freedom; CFI = comparative fit index; TLI = Tucker-Lewis index; RMSEA = root mean square error of approximation; CI = confidence interval.

### Concurrent evidence of validity

Table 7 demonstrates the concurrent evidence of validity for the Chinese BREQ-3 by regressing the global and subscale scores to their theoretically related constructs. When including the amotivation, controlled motivation, and autonomous motivation as the only predictors, amotivation and controlled motivation positively predicted competence, PA, and NA. In addition, controlled motivation positively predicted autonomy and relatedness. Autonomous motivation positively predicted autonomy, competence, relatedness, PA, and negatively predicted NA. This model explained a substantial portion of the variance for autonomy (55%), competence (55%), relatedness (37%), PA (50%), and NA (35%). Adding specific types of motivation into the model resulted in a slight improvement in the explained variances (ΔR^2^ = 0%, 2%, 1%, 0% 2% for autonomy, competence, relatedness, PA, and NA respectively).

**Table 7:** Standardized coefficients and explained variances for types of motivation regressed on covariates.

Covariates	Amoti	CM	AM	R^2^	Amoti	CM	AM	Exter	Intro	Ident	Integ	Intri	R^2^
***Autonomy***	-0.002	0.075**	0.736***	0.55	-0.038	0.109***	0.709***	0.009	-0.010	0.069	0.078	0.143**	0.55
***Competence***	0.113***	0.143***	0.715***	0.55	0.089**	0.144***	0.726***	0.014	0.003	-0.094	0.058	0.039	0.57
***Relatedness***	-0.012	0.076*	0.600***	0.37	-0.035	0.080**	0.586***	0.058	0.016	0.142**	0.071	0.039	0.38
***PA***	0.124***	0.149***	0.678***	0.50	0.111***	0.151***	0.645***	0.040	0.052	0.128**	0.097	0.147**	0.50
***NA***	0.437***	0.386***	-0.104**	0.35	0.434***	0.362***	-0.085*	0.151**	0.062	0.067	0.011	-0.122**	0.37

Table notes Amoti = amotivation; CM = controlled motivation; AM = autonomous motivation; Exter = external regulation; Intro = introjected regulation; Ident = identified regulation; Integ = integrated regulation; Intri = intrinsic motivation; PA = positive affect; NA = negative affect;

* = p <.05;

** = p <.01;

*** = p <.001.

### Reliability

Table 4 displays the composite omega(*ω*_c_), hierarchical omega (*ω*_h_), ECV, I_ECV, and PUC for different types of motivation based on the BESEM model. All motivation factors demonstrated satisfactory composite omega (*ω*_c_ >0.80). While the controlled motivation, autonomous motivation demonstrated satisfactory hierarchical omega (*ω*_h_ >0.50), the external regulation, introjected regulation, identified regulation, integrated regulation, and intrinsic motivation subscales had poor hierarchical omega (*ω*_h_ <0.50). The ECV for the controlled motivation was less than 0.85 and was greater than 0.85 for the autonomous motivation. The I_ECV values for all the items under the controlled motivation were less than 0.85 (except for item 18), whereas the I_ECV values for autonomous motivation items were above or close to 0.85 (except for item 13). The PUC was relatively low for the controlled motivation and high for the autonomous motivation.

## Discussion

The purpose of this study was to investigate the psychometric properties of the Chinese BREQ-3. The study found that the BESEM model fitted the Chinese BREQ-3 better than other alternative models. While all the items loaded on the corresponding controlled motivation and autonomous motivation factors, the items on the external motivation and introjected regulation subscales kept loading on their specific factors, but the items from the identified regulation, integrated regulation, and intrinsic motivation subscales no longer loaded onto or experienced a substantial drop in factor loadings on their specific factors. The BESEM factor structure showed good measurement invariance between men and women. The amotivation and controlled motivation showed concurrent validity evidence with NA, whereas the autonomous motivation showed excellent concurrent validity evidence with all the covariates. All the factors exceeded acceptable composite omega criteria. The controlled motivation showed satisfactory hierarchical omega value and the autonomous motivation demonstrated excellent hierarchical omega. The ECV and PUC values of the controlled motivation and the I_EVC values for the items under controlled motivation supported controlled motivation as a multidimensional construct. The ECV and PUC values of the autonomous motivation and the I_EVC values for the items under autonomous motivation supported autonomous motivation as a unidimensional construct.

Neither the CFA nor the BCFA models adequately demonstrated model fit. The misfit was caused by some items failing to associate with their specified factors when factor cross-loadings were permitted. Cid et al. [13] found a similar lack of model fit while examining the 24-item Portuguese BREQ-3 using the CFA model. After eliminating one item from each subscale, the 18-item Portuguese BREQ-3 showed a satisfactory fit. The present study took a different approach to improve model fit by slightly allowing factor cross-loadings (as close to 0 as possible) and introducing two general motivation factors to account for overall item covariations. Taken together, these two investigations demonstrated that either the item pools or the factor structure of the existing BREQ-3 should be modified to exhibit adequate construct validity evidence.

The ESEM model revealed that all of the items under the integrated regulation subscale were related to the identified regulation and intrinsic motivation subscales. The BESEM model further demonstrated that the covariances between items under the identified regulation, integrated regulation, and intrinsic motivation subscales can be sufficiently modeled by an autonomous motivation factor with minimal unexplained information remaining. Similar results were drawn in a validation study of Chinese BREQ-2 for middle and high school students [24], where the identified regulation and intrinsic motivation subscales were not distinguishable from each other. These findings suggest that the distinct conceptualizations of identified regulation, integrated regulation, and intrinsic motivation, as proposed by SDT [1, 15], is debatable when being applied to Chinese college students. This population tends to conceptualize these three types of motivation as a general autonomous motivation.

One of the advantages of a bi-factor model is that it can help researchers decide whether an instrument should be regarded as unidimensional or multidimensional by using a single or multiple overriding factor(s) to capture the covariances shared by items from different specific factors. This property is particularly advantageous when applied to SDT-based instruments, since motivation can be divided into more specific types as well as broader types [1, 15]. By noting the factor loadings of the items in the BESEM model, the controlled motivation factor captured some of the common variance for the external regulation and introjected regulation, and the items under these two subscales still loaded onto their specific factors. The autonomous motivation factor captured virtually all the common variance shared by the autonomous types of motivation (identified regulation, integrated regulation, and intrinsic motivation). These findings indicate initial evidence that using a single controlled motivation factor may not be sufficient to represent the external regulation and introjected motivation, but a single autonomous motivation can adequately represent identified regulation, integrated regulation, and intrinsic motivation.

It is worth noting that item 19 showed extremely abnormal factor loading in all the models compared to other items under identified regulation factor. In the BESEM model, this item showed a substantial low loading on the autonomous motivation factor and a substantial high loading on the controlled motivation factor. This finding indicates that participants may perceive item 19 as a more controlling rather than more autonomous measure of exercise motivation. Interestingly, item 19 was ordered as item 17 in BREQ-2. Many previous studies that examined the validity evidence of BREQ-2 have found this item problematic and decided to discard it to improve the overall model fit [24, 26]. Although item 19 did not affect the overall model fit in this study due to the allowance of cross-factor loadings, this item should not be included when calculating any factor scores for the Chinese BREQ-3.

Consistent with previous researchers who reported that the inter-factor correlations between identified regulation and intrinsic motivation in BREQ-2 were exceedingly high [18, 24, 62, 63], we found that the scores derived from the identified regulation, integrated regulation, and intrinsic motivation subscales were highly correlated in the CFA model and showed a substantive decrease in the ESEM model. Asparouhov and Muthén [34] argued that ESEM tends to provide more accurate estimates of factor correlations even when small cross-loadings are present. The suppression of cross-loadings in the CFA model would lead to the overestimation of factor correlations because it is the only way in which these cross-loadings can be expressed [33]. SDT assumes that different types of motivation are correlated along the same motivational continuum, which provides theoretical insight into the importance of considering item cross-loadings when considering the underlying structure of SDT-based instruments in order to obtain accurate factor correlations.

Configural, metric, scalar, and strict invariance of the BESEM structure of Chinese BREQ-3 across gender were established, which is necessary for accurate estimations of gender differences in factor means [64]. Based on this finding, researchers interested in the relationship between exercise motivation and other variables can safely use gender among individuals who identity as men and women as a moderator, at least for this Chinese population.

Amotivation and controlled motivation positively predicted competence and PA. In addition, controlled motivation positively predicted autonomy and relatedness. These findings did not align with SDT and findings from previous studies [20, 62]. However, given the large sample size of this study and relatively low standardized coefficients (from 0.075-0.149), these significant relationships should be considered as the result of overpower. The substantially higher coefficients when using amotivation and controlled motivation to predict NA demonstrated the concurrent evidence of validity for these two factors. The autonomous motivation factor demonstrated excellent concurrent validity evidence as it positively predicted autonomy, competence, relatedness, PA, and negatively predicted NA. Following the inclusion of the specific motivation factors, the increase of explained variance of the covariates was minimal. The specific types of autonomous motivation only provided a minimal amount of additional information in the prediction of covariates, suggesting redundant conceptualization of these motivation factors.

The composite omega and hierarchical omega were used to indicate the factor score reliability. The composite omega for all factors was relatively high, suggesting that a large amount of variance of the unit-weighted total score was due to general and specific types of motivation. The hierarchical omega of the controlled motivation indicates that among all the reliable sources explaining the score variance, the controlled motivation itself is the predominant source of explanation (*ω*_h_ = 0.63). However, there is 25% (*ω*_c_ (0.88)—*ω*_h_ (0.63)) of the reliable source that comes from specific motivation factors. The hierarchical omega of the autonomous motivation indicates this factor itself can explain virtually all the reliable variance among items (*ω*_h_ = 0.92), Only 3% (*ω*_c_ (0.95)—*ω*_h_ (0.92)) of the reliable source comes from specific motivation factors. Similar to a previous study [24], which found a three-factor structure (external regulation, introjected regulation, and autonomous motivation) should be used for Chinese BREQ-2, our findings provided strong evidence in which calculating a single controlled motivation score may not be appropriate to adequately represent external regulation and introjected regulation, whereas a single autonomous motivation score can adequately represent identified regulation, integrated regulation, and intrinsic motivation.

The ECV, I_ECV, and PUC values were calculated to examine the dimensionality of the Chinese BREQ-3. The ECV for controlled motivation (0.54) was substantially less than 0.85, suggesting that the factor scores for external regulation and introjected regulation should be calculated separately rather than together (multidimensionality). At the item level, the I_ECV value indicates that the controlled motivation factor did not adequately explain the common variance for any of the items (except for item 18). The ECV of the autonomous motivation factor was higher than 0.85, which indicates the redundancy of conceptualizing identified regulation, integrated regulation, and intrinsic motivation since it is essentially a unidimensional construct. The I_ECV for the items on the autonomous motivation factor were all higher or close to 0.85 (except for item 13), indicating that the autonomous motivation contributes a substantial amount of information to explain the common variance shared among these items. The PUC value of the controlled motivation factor was less than 0.80, according to the criteria (PUC <0.80, ECV >0.60, and *ω*_h_ >0.70), instead of calculating a single controlled motivation score, the score for external regulation and introjected regulation should be calculated separately. Although the PUC value for autonomous motivation was also less than 0.80, a single autonomous motivation score should be calculated given the high value of hierarchical omega and ECV for this factor.

This study has some limitations that should be considered. First, this sample included primarily freshman and sophomore students. The study’s findings are only generalizable to this subpopulation of collegiate students and excludes competitive athletes. To ensure ecological validity, future research on the psychometric quality of the Chinese BREQ-3 should recruit students from a diverse range of grades, as well as individuals of diverse ages, occupations, and socioeconomic backgrounds. Second, the number of students was not evenly distributed across men and women groups in this study (35% for men, 65% for women). The unequal sample sizes likely reduced the power in factorial invariance tests, potentially impacted the ability to detect noninvariance [53]. Future researchers should consider using subsampling method [65] to address this potential issue. Third, although the authors strictly followed standard criteria to translate BREQ-3 into Chinese, the content validity was not assessed for the Chinese BREQ-3. Future studies should use established assessment method (Aiken’s V) to quantify the content validity evidence for Chinese BREQ-3. Fourth, potential social desirability may bias the results of this study since no measure was used to deal with this potential issue when participants completed questionnaire online.

## Conclusion

We conclude that the current version of Chinese BREQ-3 has demonstrated adequate evidence of validity and reliability based on a bi-factor structure. We recommend calculating amotivation, external regulation, introjected regulation, and a single autonomous motivation score (excluding item 19) when using Chinese BREQ-3.
